# The Effects of Antioxidants and Pulsed Magnetic Fields on Slow and Fast Skeletal Muscle Atrophy Induced by Streptozotocin: A Preclinical Study

**DOI:** 10.1155/2023/6657869

**Published:** 2023-11-13

**Authors:** Bora Tastekin, Aykut Pelit, Tugce Sapmaz, Alper Celenk, Muhammed Majeed, Lakshmi Mundkur, Kalyanam Nagabhushanam

**Affiliations:** ^1^Department of Biophysics, Faculty of Medicine, Cukurova University, Adana, Türkiye; ^2^Department of Histology and Embryology, Faculty of Medicine, Cukurova University, Adana, Türkiye; ^3^Sami-Sabinsa Group Ltd., 19/1 & 19/2 I Main, II Phase, Peenya Industrial Area, Bangalore, India; ^4^Sabinsa Corporation, 20 Lake Drive, East Windsor, New Jersey, USA

## Abstract

**Results:**

Our findings suggest that antioxidants and PMF may alleviate impaired protein synthesis and degradation pathways in skeletal muscle atrophy. PTS showed a positive effect on the anabolic pathway, while RSV and PMF demonstrated potential for ameliorating the catabolic pathway. Notably, the combination therapy of antioxidants and PMF exhibited a stronger ameliorative effect on skeletal muscle atrophy than either intervention alone.

**Conclusion:**

The present results highlight the benefits of employing a multimodal approach, involving both antioxidant and PMF therapy, for the management of muscle-wasting conditions. These treatments may have potential therapeutic implications for skeletal muscle atrophy.

## 1. Introduction

Numerous studies have demonstrated the potential of resveratrol (RSV) to inhibit protein degradation and mitigate muscle loss [1–3]. Its anticachectic effect is mediated by the inhibition of nuclear factor kappaB (NF-*κ*B) activity and the downregulation of tripartite motif-containing 63 (*TRIM63*), also known as muscle ring finger 1 (*MuRF1*) expression [4]. RSV has also been shown to upregulate the phosphorylation of protein kinase B (Akt), p70 ribosomal S6 protein kinase (*p70S6K*), mammalian target of rapamycin (mTOR), and eukaryotic translation initiation factor 4E- (eIF4E-) binding protein 1 (*4E-BP1*) in TNF-*α*-induced atrophy [5]. This compound can alleviate muscle atrophy by activating Akt/mTOR/FoxO1 signaling pathways and downregulating the expression of F-box only protein 32 (*FBXO32*), also known as muscle atrophy F-box (*MAFbx*), and *TRIM63* [6]. Resveratrol exhibits remarkable antioxidant activity, acting as a potent scavenger of reactive oxygen species (ROS) and effectively protecting cells and tissues from oxidative damage caused by free radicals, as supported by numerous studies [7, 8].

Pterostilbene (PTS), which possesses anticancer, anti-inflammatory, antioxidant, and analgesic properties, has also been investigated in experimental studies in rats [9]. Although PTS shares many pharmacological similarities with RSV, it may be more advantageous due to its two methoxy (-OCH3) groups and greater lipophilicity, which facilitate its ability to penetrate cell membranes and increase its bioavailability and potency [10, 11].

**Figure 1:**
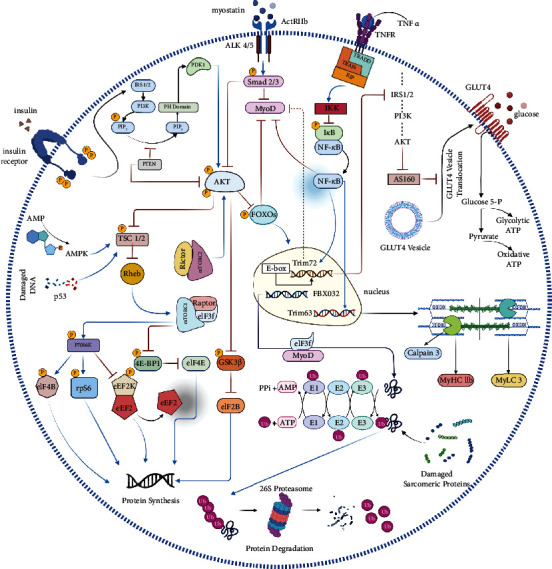
Multifaceted signaling pathways governing skeletal muscle atrophy and hypertrophy: a systematic overview. The PI3K/Akt/mTOR, extracellular insulin, and insulin-like growth factor-1 pathways are essential for protein synthesis in skeletal muscle. Upon activation, PI3K converts PIP2 to PIP3 with phosphorylation, which activates PDK1, leading to the phosphorylation and activation of Akt. Subsequently, p-Akt phosphorylates and activates mTOR, which induces protein synthesis via subpathways. Akt also phosphorylates and inhibits 4E-BP1, which plays a negative regulatory role in protein synthesis, and activates p70S6K by phosphorylation. FoxOs, when active, upregulate FBXO32 and TRIM63, which are involved in muscle atrophy. Upregulated FBXO32 ubiquitinates and degrades its substrates, and similarly, TRIM63 ubiquitinates and degrades sarcomeric proteins, leading to muscle atrophy. Myostatin inhibits Akt and MyoD, a transcription factor involved in the induction of protein synthesis. TNF-*α* inhibits NF-*κ*B and downregulates TRIM63. In the case of GLUT4, p-Akt suppresses AS160, which inhibits the translocation of GLUT4 vesicles, facilitating glucose uptake. Lastly, the ubiquitin proteasome system plays a crucial role in protein breakdown via E1, E2, and E3 enzymes. The ZNF216 receptors in the 26S proteasome system recognize Ub proteins and transfer them to the proteasome. Note that in the figure, activation of proteins is depicted by blue arrows, while inhibition is represented by red blunt-tipped arrows.

Pulsed electromagnetic fields (PEMFs) have been shown to significantly enhance molecular, cellular, and tissue functions without any apparent side effects, making them an attractive noninvasive therapy [12–14]. Nonthermal electromagnetic fields (EMFs) could potentially modify the cascade of biological processes involved in tissue growth and repair by directly impacting ion binding and/or ion transport [15]. Magnetic fields can also induce vibrations in charged ions, which may activate insulin release by exciting calcium ions in beta cells through frequency oscillation [16, 17]. These findings suggest that PEMFs may hold promise as a safe and noninvasive therapeutic modality for a range of clinical conditions. Skeletal muscle atrophy and hypertrophy are complex processes that involve the coordination of numerous molecules through intricate signaling pathways (Figure 1). The anabolic pathways associated with skeletal muscle protein synthesis rely on the positive actions of insulin/insulin-like growth factor 1- (IGF1-) PI3K-Akt/PKB-mTOR [18], while myostatin-Smad2/3 plays a negative regulatory role [19]. Conversely, the catabolic pathway responsible for protein degradation is mediated by TNF-*α* and NF-*κ*B [20]. Disruptions in the levels of key proteins involved in these pathways can upset the balance between protein synthesis and degradation, leading to diabetic skeletal muscle atrophy [21, 22].

Therefore, in this study, we aimed to investigate the effects of antioxidants, pulsed magnetic field (PMF), and combination therapy on skeletal muscle atrophy pathways. We utilized various techniques, including gene expression analysis, immunoreactivity, target protein density determination, skeletal muscle tissue morphology examinations, measurement of biomechanical parameters, and a treadmill performance test, to evaluate the effects of these treatments. A comprehensive exploration of the upstream and downstream targets of key proteins in the anabolic and catabolic pathways and their interactions promises to provide valuable insights not only into the therapeutic interventions used in this study but also into the mechanism of action and applicability of other alternative treatment modalities for diabetic skeletal muscle atrophy.

## 2. Material and Methods

### 2.1. Experimental Animals

**Figure 2:**
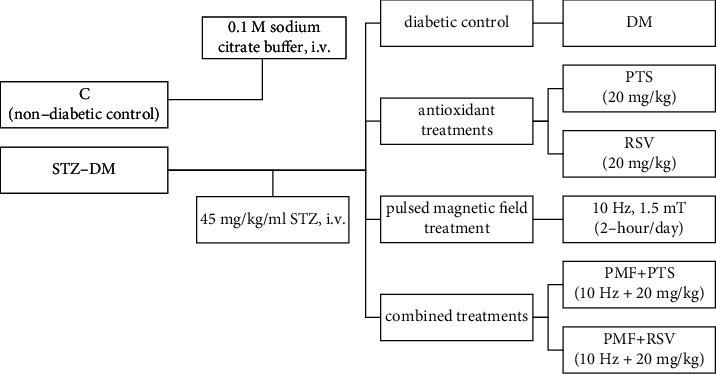
Schematic presentation of the experimental groups. The rats were categorized as either healthy or diabetic at the beginning of the study. The healthy rats formed the control group and received a single dose of 0.1-molar cold citrate buffer. On the other hand, the diabetic rats were divided into two groups, diabetic control and treatment groups, after experimental diabetes was induced by administering 45 mg/kg/ml streptozotocin (STZ). The 5-week experimental period was regularly followed throughout the study. Treadmill tests were conducted with a randomly selected subset of 5 animals from each group. The performance of animals in both the control and treatment groups was evaluated in terms of fatigue duration and distance covered.

Seventy male Wistar albino rats aged 2-3 months and weighing 360 ± 20 g were divided into seven groups of ten animals each, as follows: group C, nondiabetic rats injected (i.v.) with 0.1 M cold citrate buffer (pH 4.5); group DM, diabetic rats induced with a single dose of 45 mg/kg/ml STZ (i.v.); group DM+PTS, diabetic rats treated with 20 mg/kg body weight per day pterostilbene (i.p.); group DM+RSV, diabetic rats treated with 20 mg/kg body weight per day resveratrol (i.p.); group DM+PMF, diabetic rats treated with a pulsed magnetic field at a frequency of 10 Hz and an intensity of 1.5 mT for 2 hours daily; group DM+PMF+PTS, diabetic rats treated with a pulsed magnetic field at a frequency of 10 Hz and an intensity of 1.5 mT for 2 hours and with 20 mg/kg body weight pterostilbene (i.p.); and group DM+PMF+RSV, diabetic rats treated with a pulsed magnetic field at a frequency of 10 Hz and an intensity of 1.5 mT for 2 hours and with 20 mg/kg body weight resveratrol (i.p.) (Figure 2).

The rats were fed standard pellet rat chow and tap water as a supplementary diet and were housed in cages with a maximum of four animals per cage. The animals were kept in a controlled environment with a 12-hour light-dark cycle and maintained at a constant temperature of 22-27°C and a humidity range of 40-60%. The experimental protocols were approved by the Cukurova University Health Sciences Experimental Application and Research Center and conducted in accordance with institution guidelines for animal care and handling documented by ARRIVE guidelines and the National Institutes of Health (NIH). Blood glucose levels and body weight were monitored on a weekly basis.

### 2.2. Streptozotocin Induction

The animals were allowed to acclimate to their environment for one week without any disturbances. Before the induction of streptozotocin (STZ, Sigma Chemicals, St. Louis, MO, USA), the animals' initial blood glucose levels and weights were measured. STZ was prepared by dissolving 45 mg/kg/ml of the chemical in 0.1 M cold citrate buffer (pH = 4.6) based on the weight of each animal. The rats were anesthetized using an anesthetic vaporizer under a mixture of 60% O_2_ and 3-4% sevoflurane (Abbott, USA), and STZ was administered intravenously (i.v.) through the tail vein.

To ensure accurate monitoring of blood glucose levels, measurements were taken 48-72 hours after STZ administration by using a blood glucose meter (Accu Chek Performa Nano, Roche Diagnostics, Mannheim, Germany). Rats with blood glucose levels ≥ 300 mg/dl were classified as diabetic.

### 2.3. Antioxidant Preparation and Application

Pterostilbene (trans-3,5-dimethoxy-4′-hydroxystilbene) and resveratrol (3,4′,5-trihydroxy-trans-stilbene) were supplied by Sabinsa (NJ, USA) with a purity of >99%, as determined by high-performance liquid chromatography (HPLC). The antioxidants were prepared fresh daily during the 5-week experimental period and administered intraperitoneally in a single dose, not exceeding a total volume of 1 ml. The amounts of pterostilbene and resveratrol, dissolved in 10% dimethyl sulfoxide (DMSO), were ≤30 mg/ml and ≤50 mg/ml, respectively. To achieve homogeneous dissolution, the antioxidants were vortexed for 5-10 min before administration. To prevent UV light damage, the vials containing the antioxidants were wrapped with aluminum foil, and the procedures were performed in a low-light room. Sterile insulin syringes were used for each application.

### 2.4. Pulsed Magnetic Field Treatment and Application

**Figure 3:**
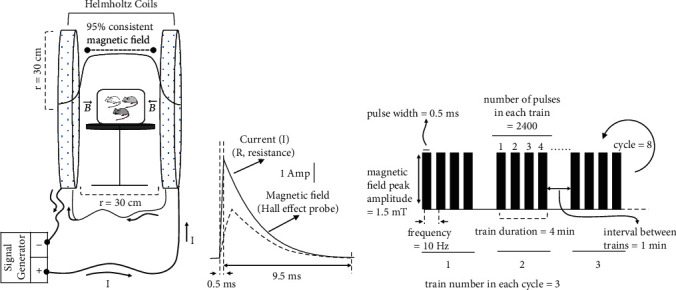
Pulsed magnetic field (PMF) treatment system and application protocol. (a) A suitably sized Plexiglas enclosure was positioned in the homogeneity region of the 95% consistent magnetic field between Helmholtz coils (*r* = 30 cm). A signal generator was used to generate a magnetic field amplitude of 1.5 mT between these coils. (b) The circuit's current (*I* = 5.2 A) was observed using an oscilloscope via a resistor (0.1 ohm) that was linked in series between the signal generator's output and the coil. The time-varying magnetic field was made up of a quasitriangular waveform with a rise time of 0.5 ms and a fall time of 9.5 ms (dashed line). (c) A schematic protocol for the pulsed magnetic field was applied to animals once daily for two hours immediately following the antioxidant injection. The peak amplitude of the magnetic field was 1.5 mT, the frequency was 10 Hz, the pulse width was 0.5 ms, and the number of pulses in each train was 2400. The train duration was 4 minutes, and the interval between trains was 1 minute. The number of trains in each cycle was 3, and the total number of cycles was 8.

To generate the pulsed magnetic field (PMF) in this study, two Helmholtz coils (HCs) with a diameter of 60 cm and positioned 30 cm apart were employed. The coils were connected to a programmable signal generator (ILFA Electronics, Adana, Turkey) to produce a magnetic field with a specific frequency and intensity. Previous researches have shown that very low-frequency magnetic field applications (≤300 Hz) offer more substantial therapeutic benefits [13, 23]. Hence, a PMF with a frequency of 10 Hz and intensity of 1.5 mT was administered to rats for 2 hours daily over 5 weeks. The peak magnetic field was measured using a Gaussmeter (FW Bell Model 6010, Sypris Company, Orlando, FL, USA) equipped with a Hall effect probe. The time-varying magnetic field was observed to have a semitriangular waveform with a rise time of 0.5 ms and a fall time of 9.5 ms. To measure the induced electric field waveform, a search coil (50 mm inner diameter, 50 turns, 30-gauge copper wire) was placed on the centerline of the HCs. The probe tips were connected to an oscilloscope (Hitachi, Tokyo, Japan) to measure the induced voltage directly. The corresponding induced electric field was observed in the form of a unipolar square wave, with peak electric fields of 0.6 V/m (0.59-0.61 V/m) in the holder between the coils. The maximum induced electric field between the coils was calculated using Faraday's law. The current in the circuit (*I* = 5.2 A) was monitored with an oscilloscope through a resistor (0.1 *Ω*) connected in series between the output of the power amplifier and the coil (Figure 3).

### 2.5. Treadmill Test

To minimize the potential impact of the treadmill test on skeletal muscle development in rats and facilitate a more accurate observation of the interventions, the test was conducted once a week with 5 randomly selected rats from each group. The rat treadmill was equipped with an adaptive system featuring an adjustable inclination of ±60° and a four-lane configuration, with each lane measuring 50 × 10 × 15 cm. In order to deliver aversive stimuli, shock grids consisting of 30 mm long bars were placed at 3 mm intervals at the back of the starting area (−*x* = 3 cm). The electric shocks could be modulated within the range of 0-10 Hz and 0-2 mA by a compatible stimulator. The motor speed of the treadmill was set to 0-90 m min^−1^ with a precision of 0.50 m min^−1^. The treadmill application protocol was carried out according to previous studies [3, 24]. Rats were allowed to acclimate to the environment by being kept on the treadmill with 0° incline at 10 m min^−1^ for 5 minutes. Then, the treadmill was set to a speed of 10 m min^−1^ at an incline of 10° for 5 minutes. The speed was subsequently increased by 2 m min^−1^ every 2 minutes until the rats reached exhaustion. The running time, speed, and distance were recorded, and the rats were lifted off the treadmill when they met the criteria for fatigue [25]; specifically, when the rats were subjected to alternating currents of 1.2 mA and a frequency of 3 Hz delivered by the shock grids attached to the back of the treadmill, they would move forward within 1.5 seconds and cling to the treadmill. If the rats failed to respond to the forward movement and remained on the grid for more than 3 seconds for the third time due to fatigue, they were considered exhausted, and the test was stopped.

### 2.6. Biomechanical Measurements

To achieve a deep state of anesthesia, the rats were administered a combination of ketamine HCl (80 mg/kg, Ketalar, Pfizer) and xylazine (20 mg/kg, Rompun 2%, Bayer). Following the induction of anesthesia, a terminal procedure was performed to ensure the animals were humanely sacrificed. This involved obtaining a blood sample via cardiac puncture, leading to rapid exsanguination and subsequent euthanasia. The right hind leg's soleus and EDL muscles were then dissected by moistening them with a Krebs solution. A force transducer was attached to a micrometer to set the optimal tension, after which a period of approximately 30 minutes was allowed for thermoregulation and equilibration. Skeletal muscles were stimulated supramaximally using 1 Hz square pulses (15-20 V) of 0.5 ms duration to record single-twitch isometric contraction responses. Contraction forces were recorded at frequencies of 10, 20, 50, and 100 Hz, respectively, using square pulses of 0.5 ms duration and voltage of 15-20 V, with a 30-minute interval between recordings. The fresh Krebs solution was used for each recording. Contraction values obtained by muscle stimulation were recorded using a force-displacement transducer (FDT 10-A 500 g, Commat, Ankara, Turkey). Parameters were determined based on single-twitch isometric contraction force (Pt, g-force), contraction time (CT, ms), relaxation time (RT, ms), and tetanic contraction force (P_o_, g-force). Biopac MP30 device, MAY organ bath (MAY WBC3044), MAY ISO-150 stimulator, and MAY water heating circulator were used for recordings. Data were analyzed using Biopac Student Lab Pro v 3.6.7.

### 2.7. ELISA and Bradford Protein Assay

Soleus and EDL muscles were collected from the right and left hind legs of rats and cut into 100-150 mg pieces. They were then cleaned with PBS solution and stored at -80°C in the Eppendorf tubes. On the day of the experiment, the tissues were rapidly pulverized using a ceramic mortar and pestle and placed in sterile borosilicate glass tubes embedded in ice-filled foam boxes. To extract the proteins of interest, the previously prepared homogenization solution, containing RIPA buffer (*w* : *v*, 1 : 10) with 50 mM Tris-HCl (pH = 7.6), 150 mM NaCl, 1% NP-40, 1% sodium deoxycholate, and 0.1% SDS (Boster Bio, USA), was added to the samples. In addition, phenylmethanesulfonyl fluoride (99%, Acros Organics, Belgium) (*v* : *v*, 1 : 100), 1 mM sodium orthovanadate (99%, Acros Organics, Belgium) (*v* : *v*, 1 : 100), and 1× protease inhibitor cocktail (Abbkine, USA) (*v* : *v*, 1 : 100) were added to prevent protein degradation. The samples were then homogenized by thorough grinding with sterile glass rods, transferred to sterile Eppendorf tubes, and centrifuged at approximately 10,000 rpm for 10 minutes. The supernatant was collected and transferred to new sterile tubes for further analysis.

pAkt (Ser473) (E2452Ra), FoxO3a (E1129Ra), MyHC IIb (E1809Ra), and MSTN (E0877Ra) were quantified using products from Bioassay Technology Laboratory (Birmingham, UK), while CAPN3 (SEC960Ra), TNF-a (SEA133Ra), NF-*κ*B (SEB824Ra), mTOR (SEB806Ra), MyLC 3 (SED425Ra), GLUT4 (SEC023Ra), and serum insulin (CEA448Ra) were quantified using products from USCN Life Science Inc. (NC, USA) following the manufacturers' instructions. Total protein concentration was determined using Bradford's solution (Serva, 5×) and bovine serum albumin (BSA, pH 7.0, lyophilic, Serva). The 5× concentration of Bradford's solution was diluted at a ratio of 2 : 7.5 with distilled water. Standard solutions were prepared by diluting the BSA reference solution with distilled water to generate the calibration curve. Subsequently, 50 *μ*l of the samples to be determined for protein concentration was added to the sample wells. Next, 200 *μ*l of Bradford's solution was added to all wells and incubated for 5 minutes at room temperature.

We utilized a microplate reader (Multiskan GO, MA, USA) to measure the absorbance values at wavelengths of 450 nm and 595 nm. Our calculations were performed through curve-fitting software (SkanIt Software 3) on a computer, and the best-fit line was determined by regression analysis. The manufacturers have established the sensitivity or lower limit of detection (LLD) of these assays as the minimum protein concentration that can be differentiated from zero.

### 2.8. RNA Isolation and Real-Time PCR (RT-PCR)

The soleus and EDL muscles were purified with fresh PBS and divided into equal portions of 60 mg, which were then placed in sterile tubes. To preserve the RNA integrity, 10-fold volume (600 *μ*l) of RNA Later solution (Biological Industries, Israel) was added to each tube, and the tubes were stored at -20°C until further processing. Skeletal muscle tissues were homogenized in nuclease-free tubes using 800 *μ*l of TRIzol reagent provided by a Zymo Research Direct-zol™ RNA Miniprep Plus kit as per the manufacturer's instructions for RNA isolation. The purity and quantity of RNA in the samples were assessed using a *μ*Drop plate (Thermo Scientific). The RNA concentration was found to be approximately 400-600 *μ*g/ml, and the RNA purity ratio (A260/A280) was calculated to be approximately 1.7.

For cDNA synthesis, a high capacity reverse transcription cDNA kit (Applied Biological Materials Inc., Richmond, BC, Canada) was used to synthesize 20 *μ*l of cDNA from 2 *μ*g RNA samples as per the manufacturer's protocol, and the cDNA samples were stored at -20°C. Primer pairs (Metabion, Germany) were designed for amplifying rat genes *FBXO32*, *TRIM63*, *FoxO3a*, *4E-BP1*, *p70S6K*, *TRIM72*, and *UbC* and are listed in Table 1. *GAPDH* (glyceraldehyde-3-phosphate dehydrogenase) was employed as a normalization control.

RT-PCR experiments were conducted using the Promega GoTaq® qPCR Master Mix Kit (Promega Corporation, Madison, WI) with a total volume of 20 *μ*l per reaction. Each reaction contained 1 *μ*l of template cDNA (1 : 4), 2 *μ*l of forward and reverse primers (10 *μ*M each), 10 *μ*l of qPCR master mix (1×), 0.2 *μ*l of carboxy-X-rhodamine (CXR) reference dye, and 4.8 *μ*l of RNase-free water. The reactions were performed using a 7500 real-time PCR system (Applied Biosystems, Singapore) with the following thermal cycling conditions: 1 cycle of initial denaturation for 3 min at 95°C, 40 cycles of denaturation for 15 s at 95°C, and annealing+extension for 1 min at 60°C. A melting curve analysis was performed from 65°C to 95°C with 1°C increments. The cycle thresholds (*C*_t_) were determined for each sample through automatic threshold analysis. Triplicate samples were run for each gene, and the *ΔΔ*C_t_ method was used to determine gene expression levels.

### 2.9. Hematoxylin and Eosin (H&E) and Immunohistochemical Staining

Muscle tissue samples were fixed in 10% neutral formalin for three days, followed by washing with distilled water to remove residual formalin. Tissue embedding was performed using the Leica TP 1020 Ototeknikon instrument following standard protocols. After blocking, 5 *μ*m-thick histological sections were obtained with a microtome and stained with hematoxylin and eosin (H&E). The sections were examined under a light microscope (Olympus BX53, Japan). Additional tissue sections were taken from the blocks for immunohistochemical analysis using primary antibodies against FBXO32 (anti-rat polyclonal antibody, PA5-76680, Thermo Fisher, MA, USA), TRIM63 (anti-rat polyclonal antibody, PA5-96226, Thermo Fisher, MA, USA), and FoxO3a (anti-rat polyclonal antibody, PA5-19519, Thermo Fisher, MA, USA).

### 2.10. Statistical Analysis

Data for each group were expressed as mean ± standard error of the mean (s.e.m.). The normality of the data was assessed using the Kolmogorov-Smirnov test. To perform statistical analysis, a one-way analysis of variance (ANOVA) with Tukey's post hoc test for multiple comparisons was employed. The level of significance was set at *p* < 0.05 for all tests.

## 3. Results

### 3.1. Impaired Glucose and Insulin Sensitivity, Total Protein Changes, and Changes in Body Weight and Muscle Weight

**Figure 4:**
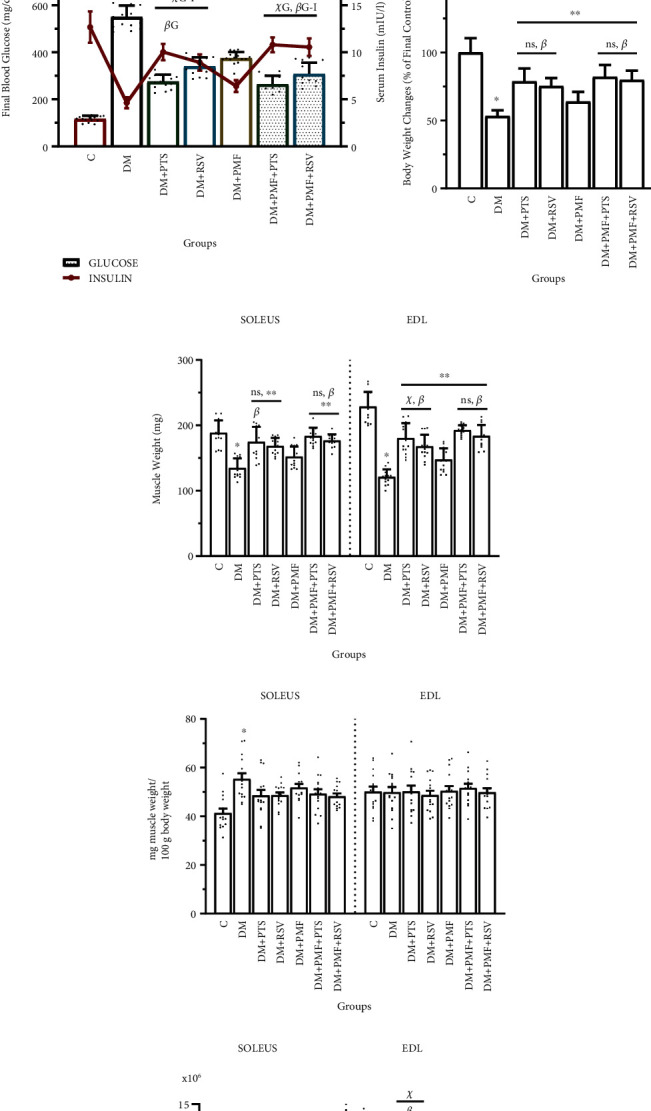
Total protein content, glucose, serum insulin levels, and changes in body and muscle weight. (a) There was no significant difference in total protein levels between the DM+PMF and DM groups (*p* = 0.11). Additionally, the PTS and RSV treatment groups did not show any significant differences in protein levels compared to each other in both soleus and EDL muscles (*p* > 0.05). (b) Final blood glucose levels (bars) and serum insulin levels (line chart) were measured. The DM+PTS group exhibited a significantly greater decrease in glucose levels compared to the DM+RSV group (*p* < 0.001), and its insulin levels increased to a greater extent than those of the DM+RSV group (*p* < 0.05). Total protein concentration in the soleus and EDL muscles was determined using the Bradford protein assay from 10 independent animals. Serum insulin levels were measured using appropriate ELISA kits from blood collected at the end of the experiment. (c) Changes in the final body weight of the rats were calculated as a percentage of the final mean weight of the control group (C) at week 5. (d) The weights of the soleus and EDL muscles were measured after the rats were sacrificed at the end of the experiment. (e) The graph illustrates the adjusted relative muscle weight of 100 g rats. (f) The cross-sectional area (CSA) of the muscles was calculated by dividing the muscle weight by the muscle length, measured prior to severing the distal tendons. The notation “ns” indicates nonsignificant differences (*p* > 0.05) between groups, “*β*” indicates significant differences (*p* < 0.05) from DM+PMF, “∗∗” indicates significant differences (*p* < 0.05) from DM, and “∗” indicates significant differences (*p* < 0.05) from C. In (b), significant differences between groups were represented by “*χ*_G_” and “*χ*_I_” for glucose and insulin, respectively. The symbols *β*_G_ and *β*_I_ were used to indicate a significant difference from the DM+PMF group for glucose and insulin, respectively. ∗_**G-I**_ was assigned to show a significant difference from C, and “∗∗_**G-I**_” was used to indicate significant differences from the DM group for glucose and insulin, respectively. All data were expressed as mean ± s.e.m., and *p* values were calculated using one-way ANOVA with the Tukey post hoc test for multiple comparisons.

We determined the total protein content in muscle tissue samples using the Bradford protein assay and evaluated the results comparatively. Notably, PMF treatment did not lead to a significant increase in total protein content in diabetic soleus muscles (*p* = 0.111 vs. DM). However, it exhibited a significant increase in total protein content in diabetic EDL muscles (*p* < 0.001 vs. DM). (Figure 4(a)). The rats' blood glucose levels were monitored weekly throughout the experiment, and at the end of the study, serum insulin levels were measured. It is well known that STZ-induced diabetic rats typically experience weight loss and elevated blood glucose levels [26, 27]. In this study, the final blood glucose levels of the diabetic rats were about fivefold higher than those of the nondiabetic control rats. All treatments were found to significantly regulate blood glucose levels, bringing them closer to the normal levels observed in the control group. Furthermore, the administration of antioxidants, PMF, and combined therapy was found to increase the serum insulin levels in diabetic rats. Although there was no significant difference between the insulin levels in the DM+PTS and DM+PMF+PTS groups (*p* = 0.320), there was a significant difference between the DM+RSV and DM+PMF+RSV groups (*p* < 0.001). Combining PMF with RSV was found to be more effective in improving insulin levels than treating with PTS alone (Figure 4(b)). These results are consistent with previous studies that have reported improved insulin sensitivity and mitochondrial formation in STZ-induced diabetic rats after administering RSV [28, 29].

The mean final body weight of rats in the diabetic group decreased by 33.51% compared to their mean initial body weight prior to STZ induction. PTS and RSV treatments demonstrated similar effects in preventing weight loss (*p* > 0.05, DM+PTS vs. DM+RSV). In contrast, the combination therapy of PMF and PTS significantly prevented 52% of the final body weight loss compared to the diabetic group (*p* < 0.001), as shown in Figure 4(c). Remarkably, upon completion of the experiment, the treatments substantially rescued muscle weight loss in the diabetic rats. The EDL muscles exhibited a greater ratio of muscle weight loss compared to the soleus muscles in the diabetic group (Figure 4(d)). The ratio of SOL muscle weight to total body weight (mg muscle/100 g body weight) in the DM group was significantly greater than that of the control group (*p* < 0.001) (Figure 4(e)). Reductions in muscle volume and mass are considered the initial signs of muscle atrophy. Muscle cross-sectional area was estimated from the muscle weight and length, with the length measurement taken before the distal tendons were cut (Figure 4(f)).

### 3.2. Antioxidants and PMF-Mediated Regulation of Key Proteins in Skeletal Muscle Atrophy Induced by STZ in Rats

**Figure 5:**
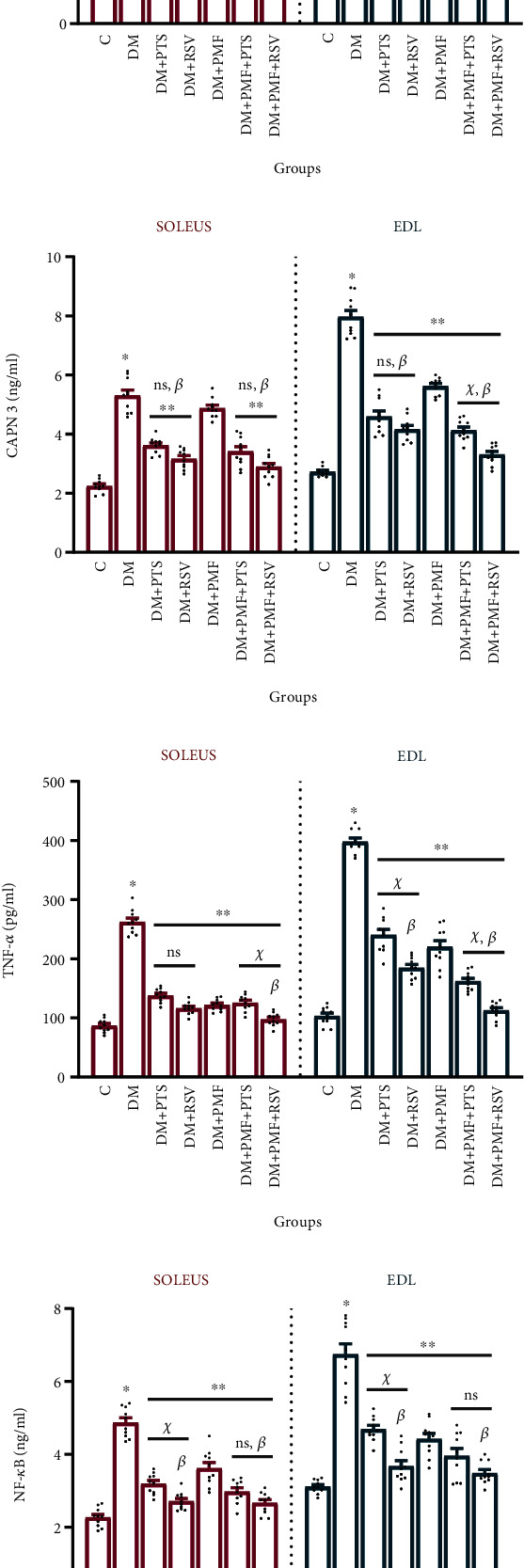
Effects of treatment on key proteins involved in protein synthesis and breakdown in diabetic rats. The study examined the effects of different treatment methods on various markers of muscle health in experimental diabetes-induced rats. (a) The level of phosphorylated Akt (pAkt) in the soleus muscle of the DM+PMF+PTS group showed a remarkable improvement, which was not statistically different from the control group (*p* = 0.514). (b) The study found no significant difference in the level of mammalian target of rapamycin (mTOR) between the DM+PMF+PTS and DM+PMF+RSV groups in the soleus muscle (*p* > 0.05), but there was a significant difference in the mTOR levels between these two groups in the extensor digitorum longus (EDL) muscle (*p* < 0.05). (c) The levels of forkhead box-O3A (FoxO3a) in both soleus and EDL muscles were significantly decreased by the applied treatment methods (*p* < 0.05 vs. DM). (d) The EDL muscles of diabetic rats showed the notable recovery after PMF+PTS application in terms of myostatin (MSTN) levels. (e) The DM+PMF group did not show significant difference in the level of calpain-3 (CAPN3) in the soleus muscles compared to the DM group (*p* = 0.185). (f) The DM+PMF+RSV group exhibited a noteworthy decrease in tumor necrosis factor-alpha (TNF-*α*) levels in both muscle types, demonstrating a significant difference compared to the DM group (*p* < 0.001). (g) The RSV alone led to a significant decrease of nuclear factor kappaB (NF-*κ*B) levels in both soleus and EDL muscles of diabetic rats when compared to the levels achieved by the administration of PTS alone (*p* = 0.034). (h) In the soleus muscles, the DM+PMF group did not show a significant difference according to the DM group in terms of myosin heavy chain IIb (MyHC IIb) levels (*p* = 0.339). (i) The increase in the amount of myosin light chain 3 (MyLC 3) in the EDL muscles of the DM+PMF+PTS group was similar enough not to differ significantly from the control group (*p* = 0.936). (j) The PTS antioxidant was found to be superiorly more effective than RSV in the EDL muscles of experimental diabetes-induced rats in terms of glucose transporter 4 (GLUT4) levels (*p* = 0.033). The ELISA analyses were conducted on the soleus and EDL muscles of 10 independent animals. The notation “ns” indicates nonsignificant differences (*p* > 0.05) between groups, “*χ*” indicates significant differences (*p* < 0.05) between groups, “*β*” indicates significant differences (*p* < 0.05) from DM+PMF, “∗∗” indicates significant differences (*p* < 0.05) from DM, and “∗” indicates significant differences (*p* < 0.05) from C. All data were expressed as mean ± s.e.m., and *p* values were calculated using one-way ANOVA with the Tukey post hoc test for multiple comparisons.

In skeletal muscle atrophy, proteins such as phosphorylated Akt (pAkt), mTOR, and forkhead box-O3a (FoxO3a) are crucial in regulating metabolic pathways [30]. Changes in these proteins can lead to the regulation of other proteins and genes that contribute to the atrophy or hypertrophy mechanism [31]. We found that all treatment groups significantly increased pAkt levels compared to the DM group. In the diabetic soleus muscle, the application of PMF+PTS showed the maximum increase in pAkt levels, while there was no significant difference (*p* > 0.05) between the groups treated with antioxidants and PMF alone in regulating pAkt levels. For the diabetic EDL muscle, PTS and RSV applications had a similar enhancing effect, but these two antioxidants significantly increased pAkt levels (*p* < 0.001) more than PMF application (Figure 5(a)).

mTORC1, which interacts with pAkt, regulates downstream and upstream signaling pathways of protein synthesis. We observed that mTOR protein levels reduced significantly with the severity of atrophy in the soleus and EDL muscles induced by STZ. There was a significant difference between the DM+PTS and DM+PMF groups (*p* < 0.001) in improving mTOR levels in the soleus and EDL muscles. However, there was no significant difference between the DM+RSV and DM+PMF groups (*p* = 0.068) in either the soleus or EDL muscles (Figure 5(b)). Our results indicate that PTS has a relatively greater potential to regulate mTOR without requiring PMF.

The phosphorylation of FoxOs by Akt reduces their tendency to enter the nucleus and prevents activation of the *FBXO32* and *TRIM63* genes [32]. FoxO3a acts as a signpost at the interface between anabolic and catabolic metabolisms and increased with STZ induction [33]. The treatments significantly reduced FoxO3a levels, which are associated with negative processes in skeletal muscle atrophy. PTS reduced FoxO3a levels in diabetic soleus muscles, while the PTS+PMF treatment could reduce the levels in both diabetic soleus and EDL muscles (Figure 5(c)).

Myostatin (MSTN), a member of the transforming growth factor-*β* (TGF-*β*) family that acts as a negative regulator in the anabolic pathway, has emerged as a new therapeutic target in the regulation of skeletal muscle mass and the treatment of muscle cell dystrophy [34, 35]. Inhibition of MSTN results in an increase in skeletal muscle mass, as it both inhibits myoblast differentiation and blocks Akt signaling [34]. All treatment groups significantly reduced the MSTN levels caused by diabetes induction. There was no significant difference between the PTS and RSV treatments in reducing MSTN levels in diabetic soleus and EDL muscles. The application of PTS+PMF brought the MSTN values closer to those of control in EDL muscles (Figure 5(d)). We assume that improvements in MSTN and TNF-*α* levels and increases in pAkt levels might result in greater FoxO phosphorylation. The increase in *FBOX32* and *Trim63* mRNA levels exhibited a proportional correlation with the inhibition of FOXO3a, as evidenced by the downregulation of its target genes. Future studies could explore FOXO3a's direct activity using reporter approaches or by assessing phosphorylation levels (e.g., Ser 253) to gain deeper insights into its regulatory mechanisms. These results suggest that targeting the FOXO interacting proteins may have a sustained inhibition of the FOXO3a pathways.

### 3.3. Skeletal Muscle Atrophy Could Be Relatively Suppressed by Downregulating Catabolic Proteins

The present study is aimed at investigating the molecular processes of skeletal muscle atrophy by examining the activity of calpain-3 (CAPN3), a protein that plays a critical role in terminating protein synthesis during skeletal muscle atrophy [36]. CAPN3 allows the exclusion of damaged myofibrillar proteins via the ubiquitin-proteasome system (UPS) [37]. As reported by Ito and Takeda [38], we also observed that CAPN3 levels were significantly increased in the diabetic group compared to the control group (*p* < 0.001). Several animal studies have shown that the activities of UPS and autophagy are elevated in T1DM, which are triggers of muscle atrophy and precursors of protein degradation [39].

Although there was no significant difference between the DM+PTS and DM+RSV groups in both soleus and EDL muscles (*p* > 0.05), RSV was slightly more effective in suppressing CAPN3 levels in the antioxidant-treated groups. In contrast, PMF significantly reduced the amount of CAPN3 only in EDL muscles (*p* < 0.001 vs. DM). The PMF+PTS and PMF+RSV treatments showed no significant difference in diabetic soleus muscles (*p* = 0.062), but the PMF+RSV significantly decreased CAPN3 levels in diabetic EDL muscles to a greater extent than PMF+PTS (*p* < 0.001) (Figure 5(e)).

In addition to CAPN3, we evaluated the levels of TNF-*α* and NF-*κ*B to assess the responses of catabolic metabolism to the treatments used in the STZ-induced atrophy model. TNF-*α* triggering activates NF-*κ*B and upregulates both MyoD and TRIM63 expressions, accelerating the atrophic process [40]. Our results demonstrated that antioxidants, PMF, and combined treatments all reduced the levels of TNF-*α* and NF-*κ*B (Figures 5(f) and 5(g)). In particular, RSV was significantly more effective than PTS at regulating catabolic proteins in soleus and EDL muscles.

Overall, our findings suggest that the combination of antioxidants and PMF treatment can regulate the molecular processes of skeletal muscle atrophy by suppressing CAPN3, TNF-*α*, and NF-*κ*B, which may have potential therapeutic implications for the treatment of muscle atrophy-related diseases.

### 3.4. The Upregulation of Myofibrillar Proteins and Glucose Transporter 4 (GLUT4)

Experimental evidence has demonstrated that *TRIM63*, a protein, plays a crucial role in binding to and activating the degradation of titin, specifically myosin heavy chain (MyHC), which is the most abundant protein in skeletal muscle [41]. We observed that the concentration of MyHC IIb was significantly higher in fast-twitch skeletal muscle fibers. In contrast, the concentration of myosin light chain 3 (MyLC 3) was significantly higher in slow-twitch skeletal muscle fibers. The use of antioxidants in combination therapy significantly improved the levels of both MyHC IIb and MyLC 3, which were reduced in diabetes. However, treatment with PMF alone did not significantly increase (*p* = 0.339 vs. DM) the amount of MyHC IIb in the soleus muscles of diabetic rats, although it did significantly increase (*p* < 0.001 vs. DM) the amount of MyHC IIb in the EDL muscles of diabetic rats. Additionally, we found that PMF significantly increased (*p* < 0.001 vs. DM) the amount of MyLC 3 in both soleus and EDL muscles of diabetic rats, which were reduced in diabetic animals (Figures 5(h) and 5(i)).

The imbalance between protein synthesis and degradation can disrupt the dynamic structure of muscles, which may contribute to diabetic myopathy [42, 43]. The presence or absence of insulin significantly impacts the balance between protein synthesis and proteolysis in diabetic skeletal muscle [44]. In T1DM, elevated blood glucose and impaired insulin response or insulin deficiency lead to decreased skeletal muscle strength and irreversible damage to the histological structure [45, 46]. In the DM group, the amount of GLUT4 in the soleus and EDL muscles of rats decreased by approximately 46.56% and 70.81%, respectively (*p* < 0.001 vs. C). After PMF+PTS administration, the amount of GLUT4 protein increased the most, by 69% in soleus (*p* < 0.001 vs. DM) and by 139% in EDL muscle (*p* < 0.001 vs. DM) (Figure 5(j)). While PTS and RSV did not significantly differ in increasing GLUT4 density in diabetic soleus muscles (*p* > 0.05 vs. DM), PTS increased GLUT4 in diabetic EDL muscles more than RSV (*p* = 0.033). PMF alone was not as effective as the antioxidants, but it continued to exert its adjuvant action and enhance the effectiveness of the antioxidants.

### 3.5. Enhancement of Immunoreactivity and Gene Expression Levels of Markers Aggravating Atrophy

**Figure 6:**
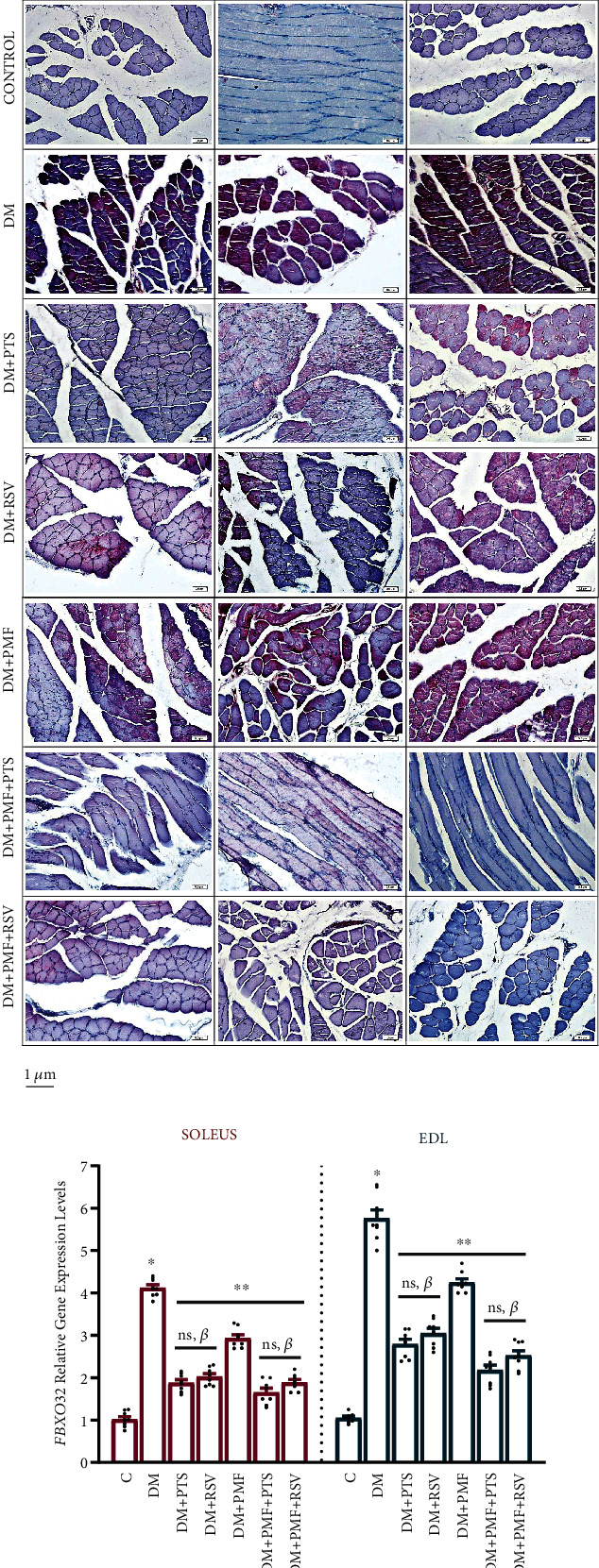
Immunohistochemical staining and gene expression of markers exacerbating diabetic muscle atrophy in soleus and EDL muscles. The immunohistochemical staining images of FBXO32, TRIM63, and FoxO3a in the soleus (a) and EDL (b) muscles were obtained from different groups. Negligible presence of markers aggravating atrophy was observed in the control group, while intense staining was detected in the DM group for all three markers. Notably, EDL muscles showed stronger staining than soleus muscles, indicating a higher degree of muscle-specific atrophy. Treatment of diabetic skeletal muscle tissues with PMF+PTS and PMF+RSV resulted in a reduction of the intense staining observed. Scale bar: 0.5 *μ*m. In addition to the immunohistochemical analyses, RT-PCR analyses were performed to determine the gene expression levels of *FBXO32*, *TRIM63*, and *FoxO3a*. (c) The expression levels of *FBXO32* in the soleus muscles were not significantly different between PMF+PTS and PTS (*p* > 0.05), or between PMF+RSV and RSV (*p* > 0.05). However, in the EDL muscles, the combined treatments of PMF+PTS and PMF+RSV showed significant differences compared to PTS (*p* = 0.01) and RSV (*p* = 0.04), respectively. (d) For *TRIM63* gene expression levels, the PTS treatment resulted in a more significant reduction in both the soleus (*p* = 0.012) and EDL (*p* = 0.020) muscles compared to the RSV treatment. (e) All treatment groups showed statistically significant differences in *FoxO3a* gene expression levels compared to the DM group (*p* < 0.05). In all gene expression analyses, *n* = 8 independent experiments were performed. The notation “ns” indicates nonsignificant differences (*p* > 0.05) between groups, “*χ*” indicates significant differences (*p* < 0.05) between groups, “*β*” indicates significant differences (*p* < 0.05) from DM+PMF, “∗∗” indicates significant differences (*p* < 0.05) from DM, and “∗” indicates significant differences (*p* < 0.05) from C. All data were expressed as mean ± s.e.m., and *p* values were calculated using one-way ANOVA with the Tukey post hoc test for multiple comparisons.

We conducted an immunohistochemical analysis to evaluate the immunoreactivities of anti-FBXO32, anti-TRIM63, and anti-FoxO3a markers involved in diabetic skeletal muscle atrophy. In control tissue samples, we observed weak activation of these markers in some areas, while in most areas, no strong staining was detected. However, in the diabetic group, we observed a pronounced presence of these markers in the cytoplasm of skeletal muscle cells, with slightly stronger activation in the EDL muscle. Upon comparing the efficacy of the antioxidant treatments, we found that treatment with PTS was more effective than RSV in reducing the immunoreactivity of these markers associated with atrophy. Although the immunoreactivities of FBXO32, TRIM63, and FoxO3a were mitigated in the soleus and EDL muscle cells of the DM+PMF group, the reductions were lower than those seen in the antioxidant groups. Remarkably, our observations revealed that treatment with antioxidants and PMF resulted in a noteworthy decrease in atrophy markers' immunoreactivity in both types of muscles, surpassing the reductions observed in the other groups, as illustrated in Figures 6(a) and 6(b).

We also analyzed the relationship between *FoxO3a* gene expression and protein levels and their correlation with *FBXO32* activation by examining *FoxO3a*'s role in activating *FBXO32*. Intriguingly, all 5-week treatments exhibited a substantial downregulation of *FBXO32*, *TRIM63*, and *FoxO3a* expression in diabetes-induced soleus and EDL muscles (*p* < 0.001 vs. DM). The upregulation of *FBXO32* gene expression in the soleus and EDL muscles of the DM group was 4.12- and 5.76-fold, respectively, compared to the control group (*p* < 0.001). The DM+PTS and DM+RSV groups showed a significant decrease in *FBXO32* expression, by 54.36% (*p* < 0.001 vs. DM) and 50.97% (*p* < 0.001 vs. DM) in the soleus muscle and by 51.56% (*p* < 0.001 vs. DM) and 47.04% (*p* < 0.001 vs. DM) in the EDL muscle, respectively, compared to the DM group. The antioxidant treatment groups were found to have reduced *FBXO32* expression significantly more than the DM+PMF group (*p* < 0.001) (Figure 6(c)).

We also found that *TRIM63* gene expression in the soleus and EDL muscles of diabetic rats increased by 3.7- and 4.83-fold, respectively, compared to the control group (*p* < 0.001). Treatment with PTS led to a 52.16% (*p* < 0.001) and 41.08% (*p* < 0.001) downregulation of *TRIM63* in the soleus and EDL muscles, respectively, compared to the diabetic control group. Similarly, treatment with RSV resulted in a 50.31% (*p* < 0.001) and 36.43% (*p* < 0.001) downregulation of *TRIM63* in soleus and EDL muscles, respectively, compared to the diabetic control group. We observed that PTS treatment was more effective than RSV at suppressing *TRIM63* gene expression in diabetic soleus (*p* = 0.012) and EDL (*p* = 0.020) muscles. Additionally, the combination of PMF and PTS treatment resulted in a greater decrease in *TRIM63* expression in diabetic soleus muscle compared to the combination of PMF and RSV treatment (*p* = 0.043) (Figure 6(d)).

The expression of *FoxO3a*, which functions as an intermediary regulator of anabolic and catabolic pathways, exhibited a substantial increase of 2.78-fold and 3.63-fold in the soleus and EDL muscles, respectively, of rats with STZ-induced diabetes when compared to the control group (*p* < 0.001). In the soleus muscle, treatment with DM+PTS and DM+RSV resulted in a significant reduction of *FoxO3a* by 38.84% and 44.96%, respectively, while in the EDL muscle, the reductions were 41.04% and 46.28%, respectively (*p* < 0.001 vs. DM). Although PTS showed greater efficacy in downregulating *FBXO32* and *TRIM63*, RSV was more effective in reducing *FoxO3a* levels in diabetic rats. The difference between DM+PTS and DM+RSV was not significant (*p* > 0.05). In diabetic soleus and EDL muscles, RSV alone was almost as effective as PMF+PTS (*p* > 0.05) in reducing *FoxO3a* expression. Furthermore, the combination of PMF and RSV significantly enhanced the efficacy of RSV in downregulating *FoxO3a* (*p* > 0.05). Overall, the combined administration of antioxidants and PMF significantly accelerated the downregulation of *FBXO32*, *TRIM63*, and *FoxO3a* in both types of muscles, as shown in Figure 6(e).

### 3.6. The Downstream Effectors of mTOR Involved in the Regulation of Protein Synthesis

**Figure 7:**
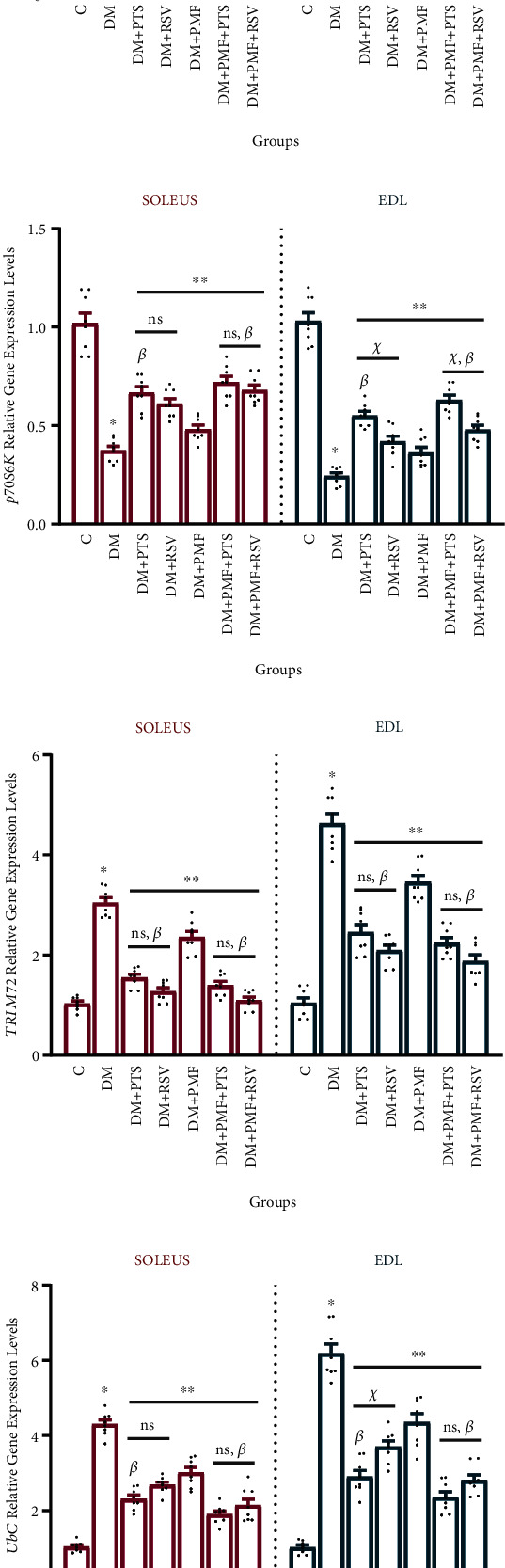
Downstream effectors of mTOR and ubiquitination. The gene expression analyses revealed differential expression of several key genes involved in muscle protein synthesis and degradation. (a) There was no statistical difference observed in the expression level of the *4E-BP1* gene between the DM+PMF group and DM group in both soleus (*p* = 0.09) and EDL (*p* = 0.56) muscles. (b) *p70S6K* showed no significant difference between the PTS and RSV treatments in the soleus muscles (*p* > 0.05), whereas RSV was significantly more effective than PTS in the EDL muscles (*p* = 0.017). (c) *TRIM72* did not exhibit any significant differences between the treatment groups in either the soleus (*p* > 0.05) or EDL (*p* > 0.05) muscles. (d) *UbC* gene expression did not show a statistically significant difference when comparing the DM+RSV group to the DM+PMF group in both the soleus and EDL (*p* > 0.05) muscles. However, in the EDL muscles, a significant difference was observed between DM+PTS and DM+RSV (*p* = 0.026). In all gene expression analyses, *n* = 8 independent experiments were performed. The notation “ns” indicates nonsignificant differences (*p* > 0.05) between groups, “*χ*” indicates significant differences (*p* < 0.05) between groups, “*β*” indicates significant differences (*p* < 0.05) from DM+PMF, “∗∗” indicates significant differences (*p* < 0.05) from DM, and “∗” indicates significant differences (*p* < 0.05) from C. All data were expressed as mean ± s.e.m., and *p* values were calculated using one-way ANOVA with the Tukey post hoc test for multiple comparisons.

The expression of *4E-BP1*, a translation initiation factor that acts as a potent translation inhibitor [47], was significantly downregulated in soleus and EDL muscles in all treatment groups except the DM+PMF group (soleus, *p* = 0.093 and EDL, *p* = 0.569) compared to the diabetic group (Figure 7(a)). The relative gene expression levels of *p70S6K*, a protein that plays an important role in skeletal muscle atrophy, were also found to be significantly reduced in both soleus and EDL muscles in all treatment groups (*p* < 0.05 vs. DM). The expression of *p70S6K* was increased by PTS in diabetic EDL muscle more than RSV (Figure 7(b)). PMF application alone did not have a significant effect on the relative gene expression levels of *4E-BP1* in diabetic soleus and EDL muscles, but it significantly increased *p70S6K* gene expression in both diabetic muscle groups (*p* < 0.05 vs. DM). This indicates that PMF may have the potential to repair diabetic-impaired protein synthesis via *p70S6K* rather than 4E-*BP1*.

### 3.7. Cell Differentiation, Ubiquitination, and Proteasome Degradation

Activation of tripartite motif-containing 72 (*TRIM72*), also known as mitsugumin 53 (*MG53*), is initiated through the myogenic differentiation- (MyoD-) mediated Smad2/3 pathway [48]. Once activated, *TRIM72* inhibits insulin receptor substrate 1 and 2 (IRS1/2) and interferes with downstream targets related to protein synthesis [49]. The findings reveal that RSV treatment significantly reduced *TRIM72* expression in diabetic soleus muscle compared to the diabetic group. The effect was evident through highly significant *p* values of 0.342 for RSV alone and 0.998 when combined with PMF. Overall, all treatment groups exhibited decreased TRIM72 expression in both soleus and EDL muscles (*p* < 0.001 vs. DM). There was no statistically significant difference between DM+PTS and DM+RSV treatments in either soleus or EDL muscles (Figure 7(c)).

Skeletal muscle is known to be affected in T1DM, and previous studies suggest that overstimulation of the ubiquitin-proteasome system (UPS) plays a significant role in muscle wasting or atrophy [50]. Our study found that PTS treatment reduced the expression of *ubiquitin C* (*UbC*) more effectively than RSV treatment, and this effect was more prominent in EDL muscles (*p* = 0.026). Moreover, PMF significantly attenuated the increased *UbC* expression in both soleus and EDL muscles of diabetic rats (*p* < 0.001 vs. DM). Combining RSV and PMF significantly decreased *UbC* levels in diabetic soleus and EDL muscles compared to RSV treatment alone, while no significant difference was observed between PTS and PTS+PMF applications (Figure 7(d)). These results suggest that different treatment methods may have varying effects on target protein density or gene expression, in addition to muscle-specific atrophy.

### 3.8. Diabetes-Disrupted Skeletal Muscle Morphology

In addition to the molecular and biomechanical analyses, we also performed hematoxylin-eosin (H&E) staining to assess the morphological structure of slow- and fast-twitch skeletal muscle tissue in diabetic rats.

**Figure 8:**
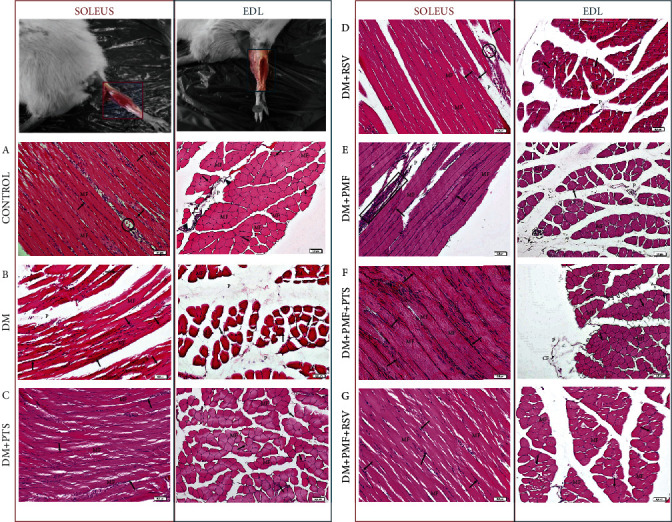
Hematoxylin-eosin staining images of soleus and EDL muscles. Hematoxylin-eosin staining was employed to obtain high-quality images of the soleus muscles in longitudinal section and the EDL muscles in cross-section. (A) The structural integrity of the muscle fibers was apparent, with individual fibers containing numerous nuclei (arrows) located peripherally. The perimysium (P), which surrounds individual muscle tissue, contained localized capillaries (circles). The nuclei of connective tissue fibers and fibroblasts (arrowheads) within the perimysium could also be distinguished. (B) The hematoxylin-eosin staining images of both soleus and EDL muscles revealed notable changes in muscle fiber organization, with the presence of pyknotic nuclei (arrows) observed in some of the fibers. In particular, the micrograph of the EDL muscle tissue showed a higher prevalence of disassembled muscle fibers compared to the control group, indicating a potential deterioration of muscle health in this region. (C) The presence of single muscle fibers with normal peripheral nuclei (arrows) indicated a mitigation of muscle fiber impairment in diabetes, with more integrated muscle bundles observed. (D) The polygonally shaped muscle fibers contained numerous hyperchromatic nuclei (arrows) located peripherally, indicating potential abnormalities in the cellular composition of the muscle tissue. Additionally, the connective tissue separating the fascicles was found to consist of capillary vessels (circles), which may play a crucial role in the delivery of nutrients and oxygen to the muscle fibers. (E) Individual muscle fibers with normal structures and peripheral nuclei (arrows) were apparent. However, the presence of inflammatory cells in the muscle tissue (squares) indicates that there may be ongoing inflammation and potential damage to the muscle tissue. (F, G) Numerous nuclei (arrows) were located peripherally along muscle fibers, suggesting a high density of muscle cells in the tissue. Additionally, the connective tissue between the fascicles was clearly visible, indicating a well-organized structure in the muscle tissue. Hematoxylin-eosin. Scale bar: 0.5 *μ*m.

In the control group, the skeletal muscle tissue of the soleus and EDL contained multiple nuclei located peripherally, and each muscle fiber had a normal morphology. The muscle fascicles, which were formed by the coalescence of muscle fibers, were surrounded by the connective tissue perimysium, which also contained large blood vessels and nerve fibers (Figure 8(A)).

In the diabetic group, we observed compromised muscle integrity, with irregularities in the formation of muscle bundles and pyknotic changes in the peripheral nuclei of muscle fibers. The structural defects were more pronounced in the EDL muscles (Figure 8(B)). However, treatment with PTS and RSV effectively regulated the muscle fibers and maintained their integrity in diabetic rats. The treatments attenuated the deterioration of the EDL muscles in diabetic rats to a similar extent as they mitigated the relatively milder degeneration of the soleus muscles (Figures 8(C) and 8(D)).

In the DM+PMF group, both muscle fiber organizations were relatively regular. However, some areas still showed pyknotic changes in the peripheral nuclei of the muscle cells (Figure 8(E)). On the other hand, the DM+PMF+PTS and DM+PMF+RSV groups showed noticeable improvement in individual muscle fibers and muscle fascicle organization compared to the diabetic group, and the number of pyknotic nuclei was significantly reduced (Figures 8(F) and 8(G)). The nuclei and cytoplasm of the muscle cells in the group DM + PMF + PTS were relatively normal, and, similar to the control group, the integrity and organization of the muscle fibers were improved.

### 3.9. Skeletal Muscle Contraction Parameters and Treadmill Test

**Figure 9:**
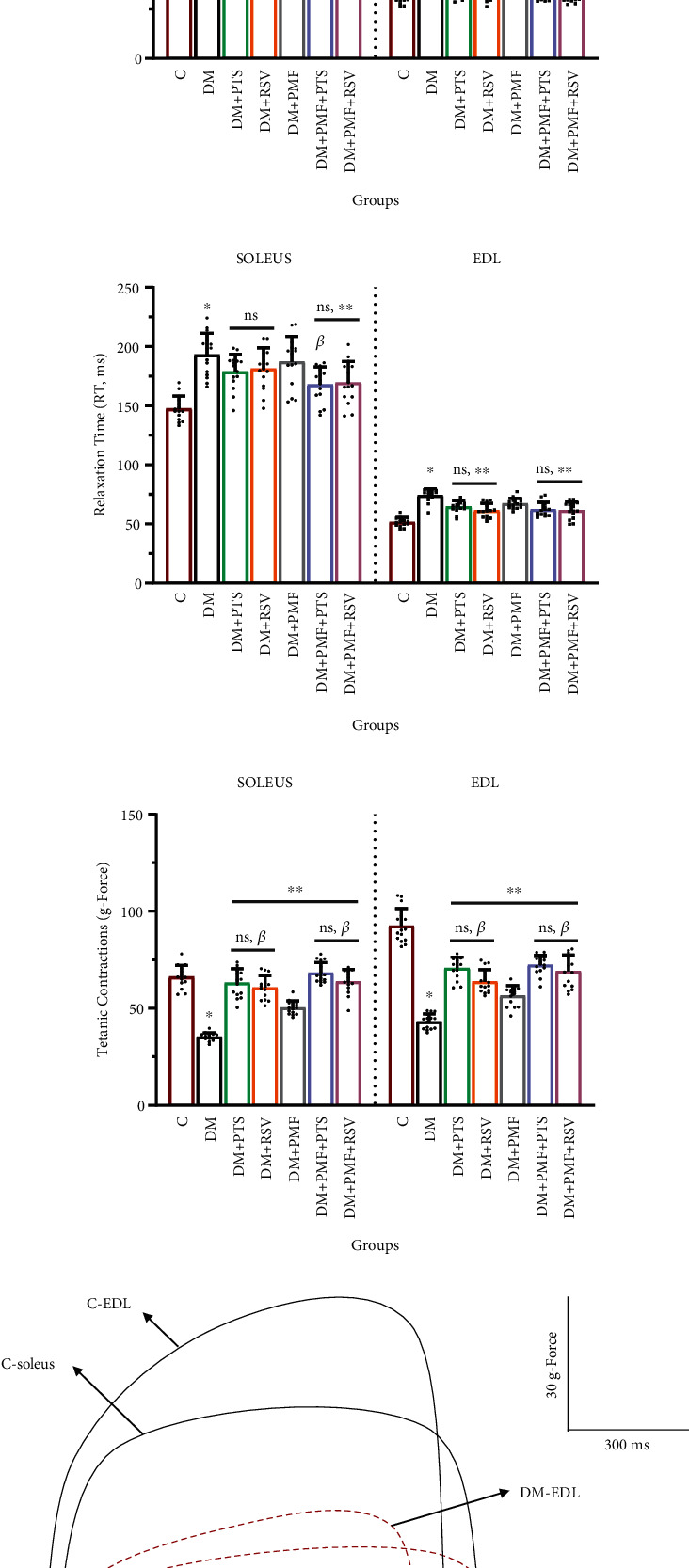
Effects of antioxidants and PMF on skeletal muscle biomechanical parameters, fatigue, and body/muscle weight in diabetic rats. (a) The maximum isometric contraction forces, measured in grams of force (g-force), generated by the soleus and extensor digitorum longus (EDL) muscles in response to single-twitch stimulation were recorded using a square wave pulse of 0.5 ms duration and a frequency of 1 Hz, with a stimulation voltage of 15-20 V. (b) A representative diagram of the isometric single-twitch contractions was illustrated to compare the force generation capacity of the soleus and EDL muscles between the diabetic (DM-soleus and DM-EDL) and control (C-soleus and C-EDL) groups. (c) The time from the onset of contraction to the peak force generation (time to peak (TTP)) was measured in milliseconds (ms). (d) The time from the peak of contraction to the end of the contraction (relaxation time (RT)) was also measured in ms. (e) The maximum tetanic contraction forces, measured in g-force, generated by the soleus and EDL muscles in response to high-frequency stimulation were recorded using a square wave pulse of 0.5 ms duration and a frequency of 100 Hz, with a stimulation voltage of 15-20 V. (f) A representative diagram of the tetanic contractions was generated to compare the force generation capacity of the soleus and EDL muscles between the diabetic (DM-soleus and DM-EDL) and control (C-soleus and C-EDL) groups. (g) Isometric contraction forces, measured in g-force, were recorded at different frequencies of stimulation, ranging from 1 to 100 Hz, using square wave pulses of 0.5 ms duration. (h) The distance covered by the rats before signs of fatigue appeared was recorded in meters (m). (i) Fatigue time was measured in minutes (min) using a treadmill test performed once a week with 5 randomly selected rats from each group. The notation “ns” indicates nonsignificant differences (*p* > 0.05) between groups, “*χ*” indicates significant differences (*p* < 0.05) between groups, “*β*” indicates significant differences (*p* < 0.05) from DM+PMF, “∗∗” indicates significant differences (*p* < 0.05) from DM, and “∗” indicates significant differences (*p* < 0.05) from C. All data were expressed as mean ± s.e.m., and *p* values were calculated using one-way ANOVA with the Tukey post hoc test for multiple comparisons.

According to the biomechanical records, control animals exhibited a 56% higher contractile force in the EDL muscles compared to the soleus muscles. In the diabetic group, both the soleus (-38.56%) and EDL muscles (-55.20%) showed a significant reduction in single-twitch contraction compared to the control group. While treatment with DM+RSV and DM+PMF did not significantly increase single-twitch contraction in the soleus muscles compared to the diabetic group, a significant increase was observed in the EDL muscles, except for DM+PMF, where the increase was not statistically significant (*p* = 0.056), as depicted in Figure 9(a). The results of the biomechanical analysis indicate that EDL muscles are more susceptible to atrophic conditions. The representative contraction curves in Figure 9(b) highlight the disparities in single-twitch isometric contraction force between the control and diabetes groups. Specifically, in the DM group, the time to peak (TTP) was longer in the soleus muscles, while the relaxation time (RT) was longer in the EDL muscles, as shown in Figures 9(c) and 9(d). These muscle-specific deteriorations may be associated with a more pronounced decrease in the binding affinity of contractile proteins and Ca^2+^ to troponin C in slow-twitch muscles compared to fast-twitch muscle fibers, as suggested in prior studies [51–53]. Furthermore, the utilization of PMF and antioxidants resulted in a significant improvement in tetanic contractions in both muscles compared to the diabetic group (*p* < 0.001), as demonstrated in Figure 9(e). The tetanic contraction force curves, displaying the measured values of the control and diabetes groups, are presented in Figure 9(f). Additionally, isometric contraction responses to different stimulus frequencies were also recorded (Figure 9(g)).

The results of the treadmill performance test revealed a significant decline in treadmill time and a 52% reduction in covered distance (*p* < 0.001) for rats in the DM group compared to the control group by the end of the 5th week. However, the administration of antioxidants and PMF effectively delayed the decline in performance and rapid muscle fatigue in STZ-induced diabetic rats (*p* < 0.05), as illustrated in Figures 9(h) and 9(i).

## 4. Discussion

In addition to the challenges and costs associated with using injectable insulin, there is evidence indicating that intensive insulin use can lead to the development of insulin resistance in individuals with T1DM [54, 55]. Research studies have revealed that insulin has the ability to suppress and inhibit UPS genes, which are responsible for protein degradation [56]. While insulin deficiency was initially believed to be the main contributor to protein degradation, subsequent studies have indicated that other signaling pathways primarily regulate atrophic states [57]. Therefore, this investigation focused on assessing the potential therapeutic benefits of antioxidants, noninvasive PMF, and their combination in the prevention of protein degradation and improvement of protein synthesis signaling pathways. The study's findings on the potential effects of combined therapy on pathways related to atrophy and hypertrophy were particularly noteworthy, as they significantly ameliorated STZ-induced muscle atrophy.

Our investigation of STZ-induced skeletal muscle atrophy revealed that the expression of *FBXO32*, *TRIM63*, and *FoxO3a* genes was more significant in fast-twitch muscle fibers (EDL) than in slow-twitch muscle fibers (soleus). This finding is consistent with previous research by Okamoto and Machida [58], who reported that *FBXO32*, *TRIM63*, and *FoxO3a* gene and protein expressions were higher in plantaris muscle fibers than in soleus muscle fibers during muscle atrophy induced by hindlimb immobilization. The *FBXO32* and *TRIM63* genes, which are activated by the E3 ubiquitin-proteasome system, are involved in protein degradation [59], and *FBXO32* is considered a crucial player in muscle proteolysis through the ubiquitin-proteasome system [60]. Although some studies have proposed that *FBXO32* gene expression is increased by inhibiting phosphoinositide 3-kinase (PI3K) activation, the exact regulatory mechanism remains unclear [61]. In general, decreased Akt activation, a common occurrence in muscle atrophy, particularly in rodent models, is associated with the activation of the FoxO/FBXO32 pathway [62]. Various muscle atrophy models have shown that decreased Akt activity leads to reduced FoxO phosphorylation, resulting in active FoxOs accumulating in the nucleus and exacerbating muscle atrophy [63–65].

In a previous study, it was observed that hindlimb unloading in rats for a period of 14 days led to an increase in the binding of 4E-BP1 to eIF-4E in gastrocnemius muscles. This suggested that 4E-BP1 plays a role in the decreased translation rate seen in muscle wasting [66]. In the present study, we found that the treatments not only increased p70S6K expression and decreased 4E-BP1 expression but also regulated pAkt and mTOR, which are typically downregulated in diabetes. This suggests that the treatments may control downstream processes in response to their increased concentrations in the medium.

Yang et al. [23] reported that 6-week PEMF application at 15 Hz, 1.46 mT, and 30 min/day significantly increased the gene expression of Akt and mTOR in the quadriceps femoris muscle of STZ-induced diabetic rats. Furthermore, the application of PEMF at these parameters resulted in a significant decrease in the levels of MSTN, activin receptor type-2B (ActRIIB), and *FoxO1*. In our study, we also applied PEMF at different parameters (10 Hz, 1.5 mT, and 2 h/day) for 5 weeks and observed significant improvements in pAkt, mTOR, MSTN, and FoxO3a levels in diabetic rats. These results are consistent with earlier reports by Yang et al. Interestingly, we also noted that pAkt levels were decreased in STZ-induced rats compared to controls in fast-twitch muscles but not in slow-twitch muscles. This observation may suggest a fiber-specific aspect of atrophy in skeletal muscle.

It is well established that MSTN and *TRIM72* are interconnected via various signaling pathways. Our results confirmed that the downregulation of *TRIM72* gene expression was linked to a reduction in MSTN protein levels after treatment. Notably, while the combination therapy was effective in controlling the atrophy of other proteins and gene expressions, no significant differences (*p* > 0.05) were observed between the combination therapy and antioxidants regarding the effects on MSTN and *TRIM72*. Previous studies have reported that *TRIM72* is a novel E3 ligase that promotes the ubiquitination and proteasomal degradation of IRS-1 in skeletal muscle [67]. In addition, calpains have a critical role in the dysregulation of proteolytic balance that occurs in muscle wasting [68]. They cleave myofibrillar proteins essential for contraction and sarcomeric structure, such as titin, nebulin, dystrophin, troponin T, troponin I, myosin, actin, and C protein [69]. Abnormally activated CAPN3 has been found to increase ubiquitin-proteasome and decrease Akt phosphorylation, leading to muscle wasting [62]. Moreover, TNF-*α*, a proinflammatory cytokine that causes protein degradation through the catabolic pathway, is responsible for increased *UbC* expression and ubiquitinated protein aggregation [70]. Our study revealed that TNF-*α* and CAPN3 protein levels, as well as *UbC* gene expression, were upregulated, and phosphorylated Akt levels were downregulated in the skeletal muscle of STZ-induced rats. This suggests that these proteins could accelerate protein degradation through a cascade-like interaction due to reduced pAkt. The treatments were effective in normalizing key proteins involved in protein degradation and improving their downstream substrates. Ono et al. [71] previously demonstrated that curcumin can attenuate skeletal muscle atrophy in STZ-induced diabetic mice by inhibiting protein ubiquitination, inflammatory cytokines, and oxidative stress. Our study showed that the reduction in NF-*κ*B levels was a sign of attenuation of catabolism, since NF-*κ*B inhibits MyoD and activates *TRIM72*. In an inflamed cell model, 5 Hz PEMF application has been found to downregulate TNF-*α* and NF-*κ*B and also tends to downregulate tumor necrosis factor-alpha-induced protein 3 (A20) [72]. In our study examining the effects of PMF on diabetic skeletal muscle atrophy, we found that the enhancement was more pronounced for catabolic pathway proteins such as TNF-*α* and NF-*κ*B than for anabolic pathway proteins.

Glucose uptake in skeletal muscle cells relies on GLUT4 channels, which are translocated from intracellular stores to the plasma membrane and T-tubules when intracellular signaling is intact [73]. Proper glucose metabolism is crucial for diabetic health, and the maintenance of metabolic continuity is partially regulated by GLUT4 protein [74]. To investigate the effects of treatments on the atrophy mechanism, we analyzed levels of GLUT4 protein. In a study of decreased GLUT4 protein expression under diabetic conditions, RSV treatment at 3 mg/kg for 7 days increased GLUT4 expression in the soleus muscles of STZ-induced rats [75]. In our study, we found that PTS increased the amount of GLUT4 protein more than RSV, which was significant in EDL muscles (*p* = 0.026) but not in soleus muscles (*p* > 0.05). The increase in GLUT4 levels may be attributed to the activation or regulation of the PI3K/Akt pathway by the treatments, which in turn inhibits Akt substrate of 160 kDa (AS160). PMF has been shown to alter the kinetics of channels, especially voltage-gated calcium (VGC) and ATP-sensitive potassium (KATP) channels [76, 77]. This promotes cellular glucose uptake by activating GLUT4 channels and facilitates insulin uptake by regulating the kinetics of insulin channel receptors, thus allowing for proper protein synthesis to occur. The rats in the DM+PMF group showed 38.22% higher insulin secretion than the DM group, indicating the potential for PMF to improve insulin secretion. Future studies will examine the functions of these channels in diabetic skeletal muscle atrophy and their impact on signaling pathways. Sakurai et al. [78] found that an increase in intracellular insulin secretion was observed when a 60 Hz/5 mT magnetic field with induction of 40 and 100 mg/dl glucose was applied to a cultured hamster-derived insulin-secreting cell line (HIT-T15) for 2 and 5 days. In our study, we observed an increase in insulin secretion that is consistent with previous studies [79–82]. This increase may be attributed to the constructive functional effects of antioxidants on beta cells. This assumption is supported by a previous study demonstrating that PTS contributes to beta-cell granulation [81].

In a mouse model of STZ-induced skeletal muscle atrophy, administration of curcumin at a dose of 1500 mg/kg/day resulted in an increase in body weight, skeletal muscle weight, and muscle fiber cross-sectional area. Moreover, curcumin reduced the expression of *FBXO32* and *TRIM63* genes in diabetic rats by decreasing the amount of ubiquitinated protein [71]. Similarly, PTS and RSV also showed promising results in rescuing skeletal muscle mass loss, suggesting the potential of antioxidants for treating skeletal muscle atrophy. Due to diabetic atrophy, skeletal muscle morphology and myofibrillar structures can also be disrupted. In our previous experimental diabetes study, electron microscopy revealed that the sarcomere structures of gastrocnemius skeletal muscles were devastated when we induced diabetes in rats with STZ [83]. Our current investigation highlights the importance of improving the expression of target proteins and genes to maintain the functionality of intracellular signaling pathways and the structural integrity of skeletal muscle during atrophy. Samir et al. [84] reported that treating type 2 diabetes mellitus (T2DM) with L-carnitine resulted in improved immunohistochemical intensity of *TRIM63*. We also observed that the immunohistochemical staining intensity of markers that exacerbate skeletal muscle atrophy in T1DM, such as *FBXO32*, *TRIM63*, and *FoxO3a*, decreased in response to treatments.

Experimental studies have indicated that skeletal muscle atrophy leads to a reduction in contractile forces and earlier onset of fatigue [85, 86]. In this study, we examined the contraction parameters of slow- and fast-twitch skeletal muscles through biomechanical recordings and demonstrated that the treatments can enhance the contraction forces of skeletal muscles. The contraction parameters of the control group were found to be consistent with those previously reported by Fraysse et al. [53], based on Ca^+2^ homeostasis in the soleus and EDL muscles. Our biomechanical analyses suggest that muscle atrophy-related muscle strength weakness may be linked to MyHC IIb and MyLC 3 component deteriorations. Furthermore, our analyses revealed that groups with elevated levels of MyHC IIb and MyLC 3 exhibited contractile responses similar to those of the control group.

We followed the muscle weakness caused by diabetes and the effects of the treatments by subjecting rats to periodic performance tests on a treadmill. In doing so, we attempted to understand the mechanism of action of the entire process from a holistic perspective. The treadmill fatigue-endurance test not only helped us assess the increase in skeletal muscle strength in rats but also indicated that the treatments used led to overall metabolic stabilization with fewer or no side effects. Our analyses revealed that FoxO-dependent atrogenes affecting contraction were more pronounced in EDL muscles and that sarcomeric proteins may be more damaged relative to MyHC IIb, MyLC 3, and *UbC* expression in these muscle types. The more pronounced loss of strength in EDL muscles may be related to impaired signaling pathways, abnormal contractions, and lower systolic Ca^2+^ concentration compared to soleus muscles.

## 5. Conclusions

In T1DM, skeletal muscle growth and development are significantly impaired [87], leading to reduced muscle mass, poor metabolic control, and a shift towards a glycolytic phenotype [88, 89]. In recent years, there has been growing interest in investigating the potential biological and medicinal properties of dietary supplements for managing diabetes. In this study, we investigated the effects of antioxidants and PMF on skeletal muscle atrophy induced by STZ in rats.

We found that both antioxidants and PMF have the potential to ameliorate the deteriorated protein and gene activities in rats with STZ-induced skeletal muscle atrophy. Notably, the examination of two different types of muscle tissue, slow- and fast-twitch, allowed for a more comprehensive and detailed interpretation of muscle-specific atrophy. Pterostilbene and resveratrol were found to be promising candidates for regulating skeletal muscle dysfunction and weakness associated with diabetic atrophy. Specifically, pterostilbene appeared to be more advantageous as a therapeutic agent.

Interestingly, our analyses showed that PMF alone did not have the same healing effects as antioxidants, but the combination of PMF and antioxidants had a greater healing effect than either treatment alone. This suggests that PMF and antioxidants may act separately on different target proteins in the anabolic and catabolic pathways and that PMF may enhance the pharmacokinetic properties of antioxidants.

In diabetic skeletal muscle atrophy, the anabolic pathway is impaired, leading to reduced protein synthesis, while the catabolic pathway is upregulated, exacerbating atrophy and accelerating protein breakdown. Our treatments hold promise for not only preventing protein breakdown by regulating the catabolic pathway but also promoting protein synthesis by enhancing the anabolic pathways. These enhancements are reflected in the structural arrangement of skeletal muscle tissue and the distribution of atrophy markers.

Future investigations will focus on further examining the proteins and genes involved in anabolic and catabolic signaling pathways, as well as the functions of ion channels important in diabetic skeletal muscle atrophy. By continuing to explore the mechanisms underlying these treatments, we hope to develop more effective interventions for managing muscle atrophy in diabetic patients.

## Figures and Tables

**Table 1:** The sequence of primers used in RT-PCR analysis.

Gene		Sequence (5′-3′)
*FBXO32*	Forward (F)	CCATCAGGAGAAGTGGATCTATGTT
	Reverse (R)	GCTTCCCCCAAAGTGCAGTA
*TRIM63*	F	GCTGCCAATCCCTACTGGAC
	R	CATGATCACTTCATGGCGGC
*FoxO3a*	F	TCTCCCGTCAGCCAGTCTAT
	R	AGTCACTGGGGAACTTGTCG
*P70S6K*	F	GGAGCCTGGGAGCCCTGATGTA
	R	GAAGCCCTCTTTGATGCTGTCC
*4E-BP1*	F	GGACCTGCCAACCATTCCAG
	R	GGGAGGCTCATCGCTGGTAG
*TRIM72*	F	CGAGCAGGACCGCACACTT
	R	CCAGGAACATCCGCATCTT
*UbC*	F	CACCAAGAAGGTCAAACAGGA
	R	GCAAGAACTTTATTCAAAGTGCAA
*GAPDH*	F	TGCACCACCAACTGCTTA
	R	GGATGCAGGGATGATGTTC

## Data Availability

The data used to support the findings of this study are included within the article.
